# Functional Clustering Drives Encoding Improvement in a Developing Brain Network during Awake Visual Learning

**DOI:** 10.1371/journal.pbio.1001236

**Published:** 2012-01-10

**Authors:** Kaspar Podgorski, Derek Dunfield, Kurt Haas

**Affiliations:** 1 Department of Cellular and Physiological Sciences and the Brain Research Centre, University of British Columbia, Vancouver, British Columbia, Canada; 2 Center for Neuroeconomics, Sloan School of Management, Massachusetts Institute of Technology, Cambridge, Massachusetts, United States of America; Mcgill University, Quebec, Canada

## Abstract

Sensory experience drives dramatic structural and functional plasticity in developing neurons. However, for single-neuron plasticity to optimally improve whole-network encoding of sensory information, changes must be coordinated between neurons to ensure a full range of stimuli is efficiently represented. Using two-photon calcium imaging to monitor evoked activity in over 100 neurons simultaneously, we investigate network-level changes in the developing *Xenopus laevis* tectum during visual training with motion stimuli. Training causes stimulus-specific changes in neuronal responses and interactions, resulting in improved population encoding. This plasticity is spatially structured, increasing tuning curve similarity and interactions among nearby neurons, and decreasing interactions among distant neurons. Training does not improve encoding by single clusters of similarly responding neurons, but improves encoding across clusters, indicating coordinated plasticity across the network. NMDA receptor blockade prevents coordinated plasticity, reduces clustering, and abolishes whole-network encoding improvement. We conclude that NMDA receptors support experience-dependent network self-organization, allowing efficient population coding of a diverse range of stimuli.

## Abstract 2

Visual experience in developing tadpoles spatially organizes neuronal receptive fields and improves network-level representation of visual stimuli.

## Author Summary

In the developing brain, sensory experience can extensively re-wire neurons, determining both their shape and function. It is thought that this early period of plasticity improves the brain's representation of sensory input. For this plasticity to actually improve coding efficiency, changes to individual neurons should be coordinated across the brain to produce a network-level functional organization. In this study, we measure such network-level changes during visual learning in developing *Xenopus laevis* (frog) tadpoles. By imaging neuronal calcium levels, we track activity in over 100 neurons simultaneously to observe changes in both single neurons and whole networks during training. We find that the network improves its representation of visual stimuli over time, by forming spatial clusters of highly connected, similarly responding neurons. Distant neurons, however, become less connected. This organization improves the ability of large groups of neurons, spanning multiple clusters, to discriminate the trained stimuli. Finally, we show that blockade of the NMDA receptor prevents this functional organization and the improvement in the network's stimulus representation. Our study shows how developmental plasticity can influence not only the proper connectivity of the visual system, but also its coding capacity.

## Introduction

The vertebrate brain exhibits intricate functional organization at many different spatial scales, from cortical microcolumns dedicated to processing specific receptive field properties, to large domains such as somatotopic maps. It is thought that this organization of neurons according to shared function optimizes efficiency and effectiveness of neural processing. During development, the structure [1],[2] and function [3]–[6] of sensory neural circuits are actively guided by both endogenous signals and environmental stimuli. However, it is not well understood how these changes lead to improved brain function.

Here we investigate how plasticity affects developing visual system performance from the perspective of sensory encoding—the representation of sensory stimuli by activity in populations of brain neurons. Neuronal responses are inherently noisy and vary across presentations of the same sensory stimulus, limiting how much information can be encoded by a single neuron [7]. To optimally encode environmental stimuli in the presence of noise [8], sensory circuits must be organized to balance redundancy, which makes network encoding less sensitive to neuronal noise, with the ability to encode a diverse range of stimuli. In the absence of noise, a given stimulus feature can be fully conveyed by a small number of neurons, and to maximize efficiency, other neurons should then encode different features. If neuronal responses are more variable, more neurons are required to reliably convey a given feature. The optimal response pattern for each neuron thus depends on the response properties of other neurons in the network and the reliability of those responses.

Encoding is also affected by neuronal interactions. For example, neuronal interactions may be organized to remove correlations from the network's input (decorrelation) [9], making the neural code more efficient, and neuronal ensembles can synergistically encode information not available from individual neurons [10]. Strategies that coordinate neuronal interactions and optimize encoding have been identified in artificial networks under various conditions [8], and encoding schemes have been described and evaluated in mature neural circuits [11]–[14]. Further studies have shown that adaptation of neuronal receptive fields [15] and correlations [16] can tune encoding in response to changes in sensory stimuli in vivo. However, little is known about how encoding schemes arise during development or how they are altered during early learning, when dynamically growing neural circuits first wire themselves together. Evaluating network encoding requires simultaneous observation of many neurons, and understanding early network refinement requires monitoring those networks over the course of learning and development.

The visual system of the *X. laevis* tadpole has been extensively studied as a model of neuronal and neural circuit development [1],[3],[4],[17]–[21]. Transparent albino tadpoles allow minimally invasive in vivo observation of rapid sensory circuit development, from differentiation [22] to mature neurons driving behavioral responses [23]. Studies in the developing brain have described mechanisms controlling large-scale circuit patterning [24], fine-scale morphogenesis [21], and rules by which synapses [25], single neurons [4],[18], and small groups of neurons [16] refine their response properties with experience. However, it is largely unknown how these developmental changes contribute to network encoding performance, or how plasticity is coordinated across neurons to produce functional large networks.

Here we use in vivo two-photon calcium imaging [3],[5],[18],[20],[23],[26] to monitor network activity and plasticity during early receptive field development in Xenopus tadpole optic tectum [27] as we train the brain to respond to a set of visual motion stimuli. Training causes stimulus-specific changes in evoked neuronal responses and increases stimulus information conveyed by neuronal firing. Decoding of network activity using computational models [28] becomes more accurate over the course of visual training. Training induces spatial clustering of receptive fields and correlations by increasing tuning curve similarity and network interactions among nearby neurons and decreasing interactions among distant neurons. Blockade of N-methyl-D-aspartic-acid type glutamate receptors (NMDARs) blocks spatially graded plasticity, and prevents decoding improvement with training. By comparing decoding in single clusters and groups, we show that increasing network performance arises from NMDAR-dependent improvement in encoding of stimulus information across clusters, while encoding within single clusters does not improve with training. We propose that NMDARs support experience-dependent functional clustering, leading to local redundancy and distant decorrelation, and promote receptive field diversity by preventing loss of underrepresented receptive fields. These results highlight contributions of network-level organization to the performance of sensory systems in vivo and identify mechanisms by which visual experience directs improvement in whole-network function.

## Results

### In Vivo Monitoring of Neuronal Firing Rates with Two-Photon Calcium Imaging

In vivo two-photon calcium imaging allows simultaneous monitoring of somatic calcium transients, induced by neuronal firing, in hundreds of neurons in the vertebrate brain [3],[5],[18],[26],[29]. We used this method to monitor correlated visually evoked responses across the optic tectum, which requires that firing-rate measurements are accurate on a single-trial basis and not averaged across trials [28]. Optical readout of calcium transients is hindered by drifting baseline fluorescence (*F*_0_), bleaching, and saturation, and involves fundamental tradeoffs between imaging area and quality of signal. Moreover, the relationship between action potentials and calcium levels is complicated by the temporal dependence of calcium concentrations on spiking history and nonlinearities in calcium influx [30]. To overcome these limitations and improve signal quality, we developed techniques for automated video segmentation to track cell boundaries on the basis of morphology and temporal pixel correlations, spatial filtering to weight the contributions of pixels within a given cell, and *F*_0_ estimation using optimal linear methods (see Methods, Figure S1). To extract firing rates from fluorescence data we employed a spike inference algorithm, which takes into account temporal dependence and nonlinearities in signal [30].

**Figure 1:**
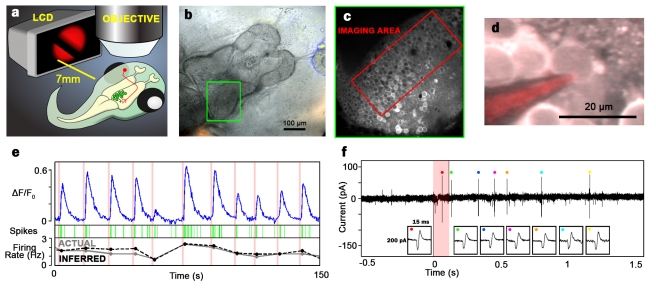
*In vivo* imaging of evoked network activity in the unanesthetized developing brain. (a) Experimental setup. Motion stimuli were presented to the left eye of awake, immobilized *Xenopus* tadpoles while imaging the right optic tectum. Neurons in the tectum (green circles) extend dendrites to receive visual input from retinal ganglion cells (red) of the contralateral eye. (b) Transmitted light image of a tadpole brain seen through the head. Green box, optic tectum. (c) Two-photon image of optical section corresponding to green box in (b). Tectum is loaded with OGB1-AM, a calcium-sensitive dye. Red box corresponds to the region of tectum monitored in our experiments. (d) Two-photon image of a patched neuron in awake tectum. (e) Simultaneous recording of somatic fluorescence (Δ*F*/*F*_0_, top) and action potentials (green) in response to full field light stimuli of varying intensity, with actual (gray) and inferred (black) firing rates in the 5 s following each stimulus. (f) Expanded voltage trace for electrophysiological recording. Pink shading marks time of stimulus. The electrical transients bounding the stimulus period are clipped. Colored dots mark individual action potentials, which are magnified in the boxes at bottom.

To assess the effectiveness of these methods for measuring single-trial–evoked firing rates in the awake brain, we performed in vivo loose seal patch clamp electrophysiological recordings to monitor action potential spiking during simultaneous calcium imaging and visual stimulation (Figures 1e and S2). We compared firing rates obtained from electrophysiological recordings to two measures of neuronal firing obtained from fluorescence data: peak Δ*F*/*F*_0_ [3] and firing rates inferred from spike inference. Though both measures showed significant correlations to actual firing, inferred firing rates outperformed peak Δ*F*/*F*_0_ in all neurons recorded (Figure S3), possibly because burst durations and interspike intervals were long (Figure 1f), resulting in imperfect summation of peak calcium currents. The relationship between inferred firing rates and actual spike counts was linear (Figure S3), showing that in vivo calcium imaging and spike inference is an effective method for monitoring firing rate fluctuations in tectal neurons.

**Figure 2:**
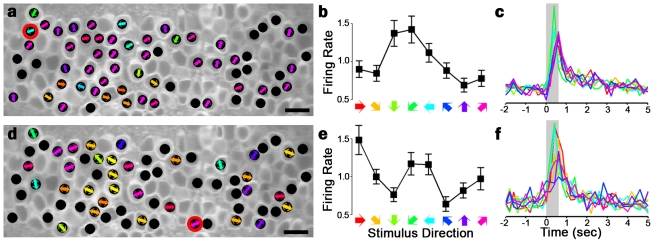
Orientation and direction responses in optic tectum. (a,d) Maps of direction and orientation selectivity in naive *Xenopus* tectum obtained through rapid two-photon imaging and firing rate inference. Stimuli were dark bars moving over a light background for 600 ms in eight directions. Black circles mark neurons that responded significantly to stimuli. Colored arrows mark preferred directions (a) and orientations (d) of neurons showing stimulus specificity. Coronal optical section, rostrum to the left. Scale bar = 20 µm. (b,e) Tuning curves of a direction- (b) and an orientation- (e) selective neuron highlighted in (a,d). Error bars denote SEM. (c,f) Average temporal response of the two neurons to each stimulus direction. Colors match those in (b,e). Gray bar marks time of stimulus presentation. All measures calculated from *n* = 48 stimulus presentations for each of eight directions (1 h).

We first used rapid two-photon imaging and firing rate inference to characterize motion receptive fields in untrained tadpoles. Motion stimuli consisted of dark bars moving over a light circular background in each of eight directions (see Methods), with low contrast so as to better detect improvements in neuronal responses with subsequent training. We found that most motion-responsive tectal neurons respond either symmetrically to pairs of opposing directions (orientation selectivity, 59.1%±5.0% of cells; mean ± standard deviation [SD]), and/or specifically to a narrow band of directions (direction selectivity, 66.3%±11.1%). Neurons responding to two opposite directions while strongly favoring one direction can show both selectivities (36.7%±9.3%). Average responses of individual neurons to each stimulus direction, called tuning curves, show varying selectivity in a topographic organization (Figure 2). These results demonstrate the effectiveness of two-photon imaging and spike inference in measuring receptive fields across a contiguous brain network in vivo.

### Tectal Network Responses to Visual Stimuli Exhibit Noise Correlations Indicating Functional Interconnections

**Figure 3:**
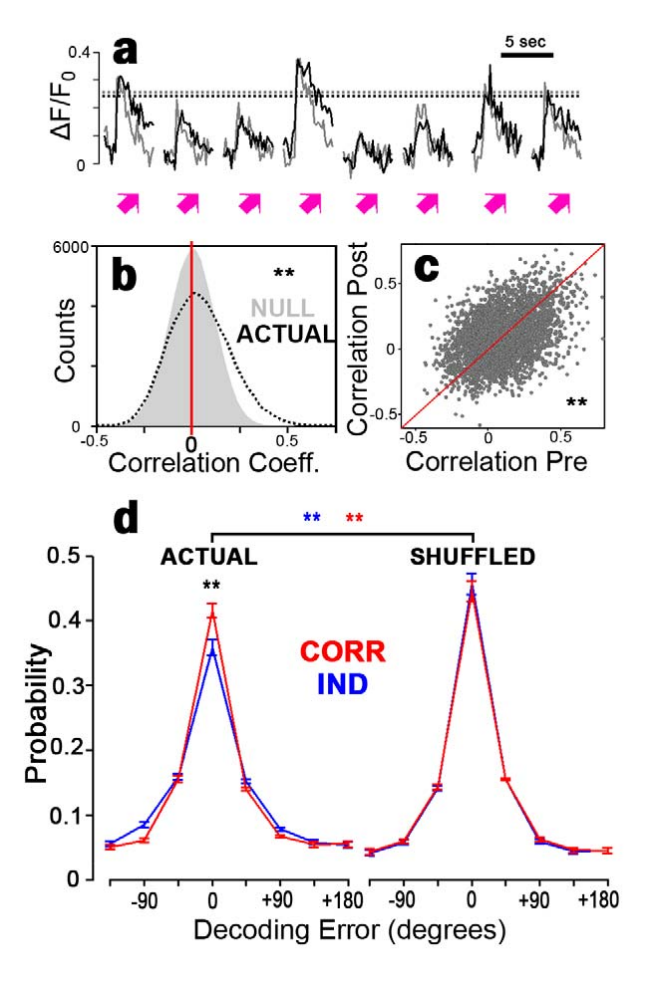
Tectal noise correlations influence network decoding. (a) Recorded responses of two neurons (black and grey) in the same tadpole to eight consecutive presentations of the same stimulus. Responses vary in amplitude around their means (dotted lines). These neurons were noise correlated: variations in amplitude were shared. (b) Distribution of measured pairwise noise correlations (black dotted e) taken over a 1-h stimulation period, and values expected if neurons were independent (gray). Noise correlations were more positive (*p*<10^−5^, *t*-test) and more variable (*p*<10^−8^; X^2^ variance test) than chance. (c) Scatterplot of pairwise linear noise correlations measured in two consecutive 30-min periods. Consecutive noise correlation measurements are correlated (*r* = 0.41, *p*<10^−8^; linear regression). (d) Distribution of decoding errors under independent and noise correlation decoding of actual response patterns (left) and with responses shuffled for each stimulus type to remove noise correlations (right). Data from seven tadpoles, 277 neurons (b,d), 384 stimulus presentations (c), 192 stimulus presentations each 30 min. Error bars denote SEM. *p<0.05; **p<0.01.

Besides the single-neuron properties described above, networks of neurons often show correlations in their firing patterns. Neurons with similar tuning curves show “signal correlations” because their firing is driven by the same stimuli [11]. Notably, real neuronal responses also show trial-to-trial deviations from their tuning curves. When these trial-to-trial deviations are shared, because of common input or interconnections, neurons are “noise correlated” (Figure 3a) [11]. Noise correlations are thus correlations in neural firing patterns that are not explained by shared receptive field properties. Noise correlations can be positive or negative, can differ across stimuli, and do not require signal correlations to be present. When trial-to-trial variability is not shared, neurons are independent.

The contribution of neural correlations to network activity patterns is difficult to determine when observing only individual neurons or small groups [28]. Effects of pairwise interactions on network encoding may only be detectable if many neurons are taken into account, and even small pairwise interactions strongly impact activity patterns when large networks are considered [31]. Thus, when neurons are significantly noise correlated, understanding network function requires observing activity in large groups of neurons simultaneously [28],[32]. Numerous studies have investigated the presence of noise correlations in vivo [11],[16],[33], their effects on encoding [8],[12],[34],[35], and the consequences of ignoring them [36]. Conclusions on these topics vary with the brain regions and response properties being studied. It is agreed, however, that the presence and impact of noise correlations determines the experimental and theoretical methods we must use to understand neural information processing.

Examining multineuronal firing patterns elicited by motion stimuli, we find that noise correlations are prominent in the awake developing tectum (Figure 3b and 3c). Noise correlation measurements were correlated over consecutive 30-min periods (Figure 3c). Noise correlations varied across stimuli (Figure S4), and may thus convey stimulus information not present in single-neuron responses (Figure S5) [37]. Noise correlations between neurons tended to have the same sign as signal correlations (Figure S6), indicating that many tectal noise correlations reflect shared errors in similarly responding neurons. These results demonstrate that tectal noise correlations can be measured with two-photon calcium imaging and may have consequences for information processing in this network.

### Tectal Noise Correlations Can Encode Stimulus Information, But Impair Overall Network Performance

Noise correlations can both help and hurt network stimulus encoding, depending on how they vary with stimuli and the response properties of neurons in the network [8],[11],[32],[35],[37]. Because noise correlations are prominent in developing tectum and are stimulus dependent, we expected that knowledge of noise correlations may be important for downstream neurons to extract all available information from network activity patterns. However, because we found that tectal noise correlations largely reflect shared errors, we expected removal of noise correlations from population activity would increase the amount of information available in those firing patterns [7],[8]. To test these predictions, we constructed two model decoders: one that takes into account pairwise noise correlations, and an optimal independent decoder, which ignores noise correlations. A decoder is a model based on a set of real network responses, which takes a second set of measured activity patterns as input and predicts the inducing stimuli [28]. Decoders thus perform the same task as downstream neurons to recover stimulus information from upstream network activity. By building decoders, we can ask two distinct questions: Regarding encoding—Would population encoding accuracy be altered if noise correlations were somehow abolished? Regarding decoding—Is knowledge of noise correlations necessary to fully decode network activity from a population response? We find that abolishing noise correlations by shuffling neurons' responses across trials of each stimulus improves accuracy of both decoders (Figure 3e). This finding confirms that encoding would improve overall if responses were uncorrelated, likely because the noise correlations we observe are largely shared errors among similarly responding neurons. Nevertheless, ignoring noise correlations in actual data significantly reduced decoding accuracy (Figure 3e). This outcome suggests that sensitivity to noise correlations would help downstream neurons to decode firing rates in this network. However, changes in neural response properties over the sampling period can make noise correlations important for decoding, even in cases where they would not be important if responses were stationary [38]. To properly evaluate the contribution of noise correlations to decoding we must thus determine whether tectal responses change with repeated stimulus exposure, and manipulate this contribution by altering neuronal interactions.

### Visual Training Induces Neural Plasticity, Improving Stimulus Encoding

**Figure 4:**
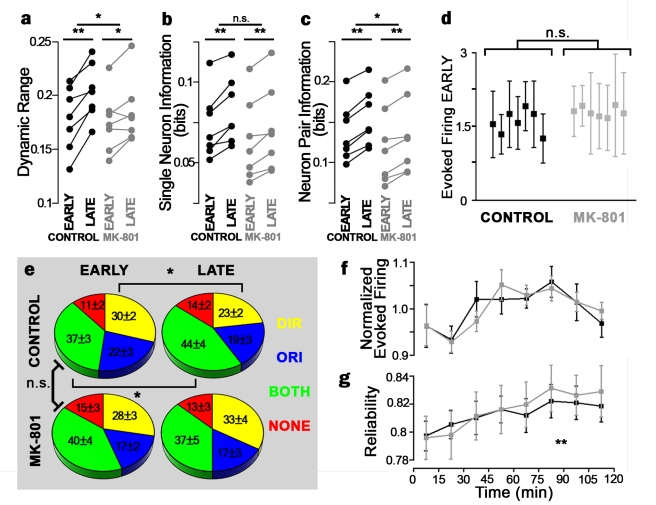
Effects of visual training on single-neuron response properties. (a) Tuning curve dynamic range, the fraction by which a neuron's firing changes in response to different stimuli during early and late epochs. (b,c) Stimulus mutual information conveyed by single neuron (b) and neuron pair (c) firing patterns. Upper asterisks denote difference in the change with treatment. Lower asterisks denote significant change across epochs (paired *t*-test). (d) Evoked firing rates in control (black) and MK-801 treated (gray) tadpoles during first hour of stimulation. Each point corresponds to a single tadpole; error bars denote standard deviation across neurons within a given tadpole. MK-801 does not acutely affect evoked firing rates (*t*-test, *p* = 0.61). (e) Proportion of neurons showing direction (yellow), orientation (blue), both (green), or neither (red) selectivity in control (top) and MK-801–(bottom) treated tadpoles, in the first (left) and second (right) hour of stimulation. Asterisks denote significant change across epochs (paired *t*-test). (f,g) Mean normalized amplitude (f) and response reliability (g) over the course of visual training (black). Reliability increased with training (ANCOVA, *p*<0.01). Neither measure was affected by MK-801 (gray) (ANCOVA, *p*>0.05). Reliability is the proportion of evoked responses with amplitude larger than the median spontaneous firing rate. Error bars denote SEM. *p<0.05.

**Figure 5:**
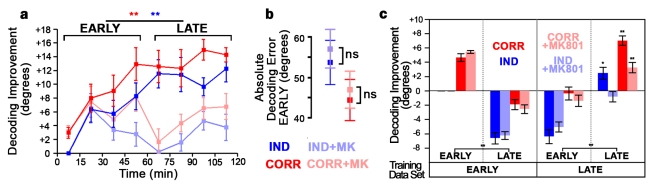
Training induces NMDAR-dependent improvement of whole-network encoding. (a) Time course of noise-correlation–based (red) and independent (blue) decoding performance. Light curves, improvement is blocked by MK-801. Bars denote early and late epochs. Decoding improvement is the decrease in decoding error relative to the independent decoder at the first timepoint. Both decoders improved from early to late epochs in control, but not MK-801–treated tadpoles (paired *t*-tests). (b) Decoding error of control (left, blue) and MK-801–treated (right, red) tadpoles over first hour of stimulation. Lighter shades denote decoding using the optimal independent decoder, darker shades mark noise correlation-based decoding. (c) Improvement, relative to the early epoch, of decoders trained on data from early (left two panels) or late (right two panels) epochs, used to decode early or late neuronal firing patterns. Performance decreased when decoding the epoch on which the decoder was not trained (center two panels; ANOVA). Asterisks in rightmost panel denote significant difference from corresponding value in leftmost panel. Error bars denote SEM. *p<0.05; **p<0.01.

During development, sensory experience drives dramatic neural plasticity [3]–[5],[18],[20],[26], but how these changes lead to improved circuit function is not understood. To investigate how stimulus encoding changes in response to visual experience, we presented tadpoles the eight motion stimuli of different directions repeatedly over 2 h. This training improved sensory responses over time, increasing dynamic range and response reliability (Figure 4a, 4f, and 4g). Training also shifted neural response properties, increasing the proportion of neurons showing combined orientation and direction selectivity and decreasing the proportion showing only direction selectivity (Figure 4e). Encoding was enhanced, evident from increased stimulus mutual information conveyed by both individual neurons and neuron pairs (Figure 4b and 4c), and improvement in both independent and noise-correlation–based decoding of whole-network activity (Figure 5a). To further demonstrate that visual experience modifies network encoding over time, we split the stimulation period into two 60-min epochs (“early” and “late”), and built decoders for each using firing statistics from either the same or the opposite epoch. Both independent and noise correlation decoding improved from early to late epochs, and decoding performance decreased when using firing statistics from the opposite epoch (Figure 5c), demonstrating that experience changes how developing brain networks encode stimuli.

### NMDAR Blockade Does Not Alter Basal Neuronal or Network Responses

NMDARs act as molecular detectors of correlations between pre- and postsynaptic firing and are known to mediate several types of functional [3],[25],[39] and structural [20],[21],[40],[41] plasticity in tectal neurons. To investigate NMDAR roles in shaping neuronal correlations and network-level encoding, we tested tadpoles treated with MK-801, a noncompetitive NMDAR antagonist. MK-801 was infused directly into the tectum and applied to tadpole bath, conditions we find to completely block NMDAR synaptic currents evoked by optic nerve stimulation in vivo (Figure S7). With calcium imaging, we first investigated the acute effects of MK-801 on neuronal firing and network performance. NMDAR blockade did not affect basal neuronal firing rates (Figure 4d), or the relative proportions of different types of motion stimulus selectivities across neurons (Figure 4e). MK-801 treatment also did not alter basal network encoding performance (Figure 5b) or neuronal reliability (Figure 4g). Previous studies have also found that NMDAR antagonism does not acutely affect tectal motion responses [4], and MK-801 does not acutely affect cortical response properties [42], or temporal properties of evoked tectal firing [19]. Consistent with these studies, we find that NMDAR currents do not contribute strongly to visually evoked responses in this system.

### NMDARs Mediate Experience-Driven Network Plasticity

To investigate NMDAR effects on experience-dependent network plasticity, we performed the previously described visual training protocol using moving bar stimuli of eight directions with tadpoles treated with MK-801. We find that distinct components of experience-dependent plasticity are NMDAR dependent and independent. In contrast to untreated tadpoles, training did not shift the proportions of different response selectivities in MK801-treated tadpoles (Figure 4e). MK-801 reduced improvement in whole-network encoding, dynamic range, and stimulus information of neuron pairs, but not in single-neuron stimulus information (Figure 4a–4c and 5a). MK-801 also blocked increases in decoding performance when the stimulation period was split into early to late epochs (Figure 5c). In fact, correlation-based decoding with MK-801 worsened from early to late epochs when decoded with each epoch's own training statistics, suggesting a strong role for NMDARs in changes to network interactions and their effects on population encoding.

Further aspects of network plasticity observed with training were NMDAR-independent. MK-801 treatment did not affect the time course of neuronal reliability or mean response amplitude (Figure 4f and 4g), and a significant portion of training-induced increases in mutual information and dynamic range remained in MK-801 treated tadpoles (Figure 4a–4c).

### Training-Induced Plasticity and Encoding Improvement Are Stimulus Specific

**Figure 6:**
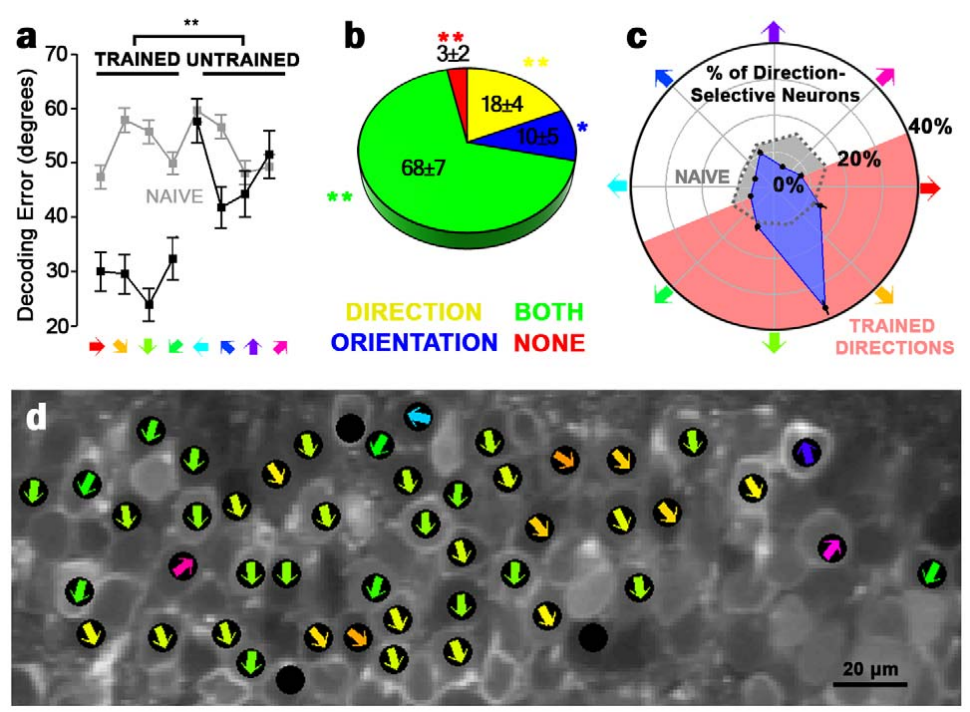
Training-induced changes are stimulus-specific. (a) Decoding error for each direction in tadpoles trained with four of eight stimuli (0–135°), using the correlation decoder. Gray, decoding error of naive control tadpoles. Training-induced decoding improvement is specific to the trained stimuli. (b) Proportion of neurons showing direction (yellow), orientation (blue), both (green), or neither (red) selectivity in tadpoles trained with four stimuli. Asterisks denote significant difference from corresponding proportion in naive control tadpoles. (c) Angle histogram of preferred directions of direction-selective neurons in tadpoles trained with four stimuli. Points are the proportion of neurons with center directions falling between adjacent stimulus directions. Pink shading indicates the trained directions. Responses strongly favored the center of the trained directions (one-sample *t*-test, *p*<10^−5^). Gray dotted line indicates preferred directions in naive control tadpoles. (d) Map of direction selectivity in a tadpole after training with four stimuli. Black circles mark neurons showing significant direction selectivity. Colored arrows mark preferred directions. Error bars denote SEM. (a–c) *n* = 3 tadpoles (152 neurons). *p<0.05; **p<0.01.

To determine whether improvements in network function are specific to the training stimuli, we trained tadpoles for 1 h with four of the eight motion stimuli (0°, 45°, 90°, 135°), followed by probing with the full eight stimuli (0°–360°), and compared network responses to trained versus untrained stimuli. Training improved decoding of the trained stimuli only for both the correlation-based (Figure 6a) and independent (unpublished data) decoders. Relative to naive tadpoles, training with four stimuli increased the proportion of neurons showing combined orientation and direction selectivity and decreased the proportion of responsive neurons showing no selectivity (Figure 6b). Among direction-selective neurons, direction of selectivity favored the center of the trained directions (Figure 6c and 6d). Dynamic range was higher in response to trained stimuli, while reliability and evoked firing were not significantly different between trained and untrained stimuli (unpublished data). These results demonstrate that training-induced changes are stimulus dependent and favor encoding of the specific visual stimuli experienced.

### Training Induces Anatomically Structured Network Plasticity

**Figure 7:**
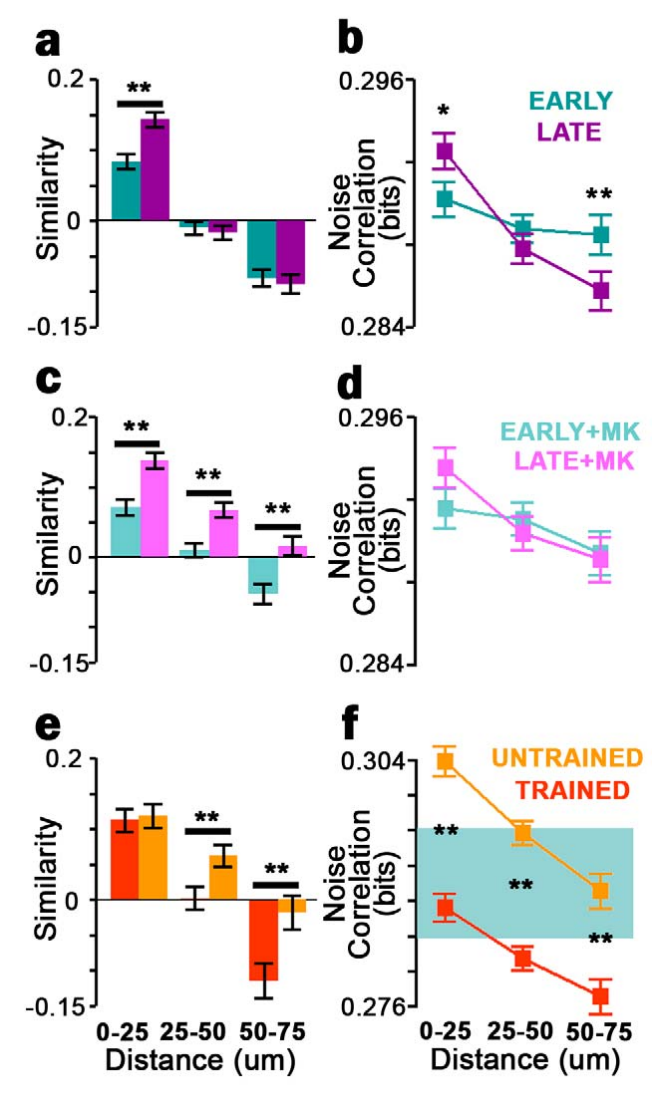
Training strengthens clustering of receptive fields and network correlations. (a–d) Tuning curve similarity (a,c) and mean noise correlation (b,d) of neuron pairs binned by spatial distance, during early (teal) and late (purple) epochs, in control (a,b) and MK801-treated (c,d) tadpoles. (e,f) Tuning curve similarity (e) and noise correlations (f) in tadpoles trained with four stimuli (0°–135°), binned by distance, in response to trained (orange) and untrained (yellow) stimuli. (f) Shaded area highlights the range of plots in (b,d). Noise correlations to untrained stimuli were significantly lower than in naive control animals (*p*<10^−5^, two-way ANOVA) and those to trained stimuli were significantly higher than in naive controls (*p*<10^−5^, two-way ANOVA). Error bars denote SEM. Control, *n* = 7 tadpoles (277 neurons), MK801, *n* = 7 tadpoles (255 neurons) (e,f) *n* = 3 tadpoles (152 neurons). *p<0.05; **p<0.01.

Imaging a contiguous population of neurons allows us to relate experience-dependent plasticity to anatomical structure [5]. Similar to visual cortex [29], optic tectum has a precise functional architecture [18], where nearby neurons exhibit similar receptive fields and thus strong signal correlations (Figure 2). We also find that nearby neuron pairs show higher noise correlations and a significant association between stimulus and noise correlation, consistent with locally shared input or direct connectivity. We tracked these measures across epochs of visual training among nearby (<25 µm), moderate (25–50 µm), and distant (50–75 µm) neurons. Tectal somata have diameters of 10–15 µm. These measures changed in a distance-specific manner as visual training improved network encoding. Visual training increased signal correlations among nearby but not more distant neuron pairs (Figure 7a). Visual training also increased nearby noise correlations and decreased distant ones (Figure 7b). Larger signal and noise correlations for nearby neurons indicate increased local redundancy with training, likely because of strengthening of shared stimulus inputs. The decrease in distant noise correlations, however, suggests that encoding strategies thought to improve mature circuit performance [8],[9], such as network decorrelation, can result from plasticity during early experience in vivo. These results show that visual training leads to anatomically structured network refinement.

**Figure 8:**
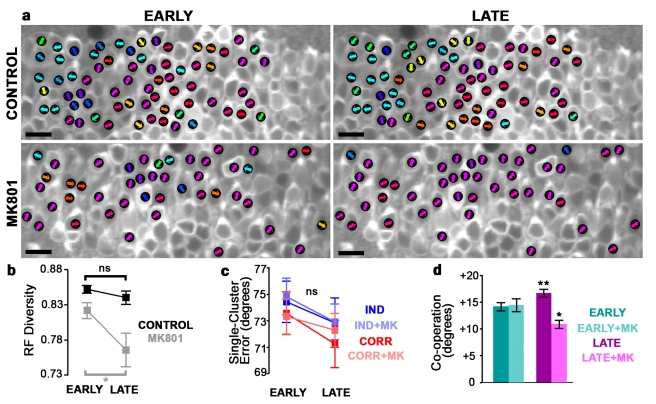
NMDAR-dependent coordination between clusters supports network encoding improvement. (a) Preferred directions of example control (top) and MK801-treated (bottom) tadpoles during early (left) and late (right) epochs. Scale bar = 20 µm. (b) Receptive field diversity across the tectum during early and late epochs, in untreated (black) and MK801-treated (gray) tadpoles. Diversity decreased with training in MK-801–treated tadpoles (paired *t*-test, *p*<0.05). (c) Mean decoding error of independent (blue) and correlation-based (red) decoding of single clusters during early and late epochs, in untreated and MK801-treated (lighter shades) tadpoles. (d) Mean decoding cooperation (decoding performance of two clusters taken together minus the maximum decoding performance of either taken alone) during early and late epochs, in untreated and MK801-treated tadpoles. Cooperation increased in control tadpoles and decreased in MK-801 treated tadpoles with training (paired *t*-tests). Number of clusters: untreated, *n* = 29; MK801, *n* = 25 clusters per epoch in seven tadpoles. Error bars denote SEM. Control, *n* = 7 tadpoles (277 neurons); MK801, *n* = 7 tadpoles (255 neurons). *p<0.05; **p<0.01.

NMDAR blockade prevented this refinement and led to degradation of fine-scale functional organization over time. Here, signal correlations were increased equally for all neuron pairs, regardless of spatial distance, reducing receptive field diversity across the tectum (Figures 7c and 8b). MK-801 also blocked training-induced changes in noise correlations (Figure 7d), suggesting that development of efficient network correlation structure is NMDAR-dependent. The loss of spatial organization we observe with MK-801 over time is consistent with lack of competition between locally represented and distant inputs in the absence of NMDAR transmission.

MK-801–induced changes in plasticity were recapitulated by training with the four-stimulus subset. Tuning curve similarity was greater over untrained stimuli than trained stimuli across moderate and distant, but not nearby, neuron pairs (Figure 7e). Networks showed strongly decreased noise correlations to trained stimuli, while noise correlations to untrained stimuli increased above levels in naive tadpoles. These results show that training with a set of stimuli affects the encoding of unpresented stimuli, and stimuli can compete in determining network connectivity (Figure 7f) [40],[43],[44].

### Coordination between Neuronal Clusters Supports Experience-Dependent Encoding Improvement

Visual training induces remarkable spatially divergent plasticity. On one hand, training-induced encoding improvement is associated with lower signal and noise correlations among distant neurons. On the other hand, local plasticity opposes this trend, increasing redundancy between nearby neurons over the course of visual training. To determine how these opposing forces contribute to overall network improvement, we grouped neurons according to receptive field so as to monitor stimulus decoding within clusters of similarly responding neurons over time (see Methods). Consistent with our measurements of tectal signal correlations, functionally defined groups showed significant spatial clustering (Figure S8). Interestingly, decoding success of single clusters did not change with training (Figure 8c), suggesting that interactions between clusters may be more important in supporting overall encoding improvement. To understand how well clusters interact to encode information, we measured intercluster cooperation, which we defined as the decoding performance of two clusters taken together minus the maximum decoding performance of either taken alone. Cooperation is high when clusters encode distinct information or encode information synergistically [10], and low when clusters encode the same information. Notably, cooperation increased with visual training in control tadpoles, while training during NMDAR blockade decreased cluster cooperation (Figure 8d). To further investigate how plasticity in neuronal interactions contributes to changes in encoding performance, we again removed the contribution of noise correlations by shuffling neuronal responses prior to decoding (as in Figure 3e). Shuffled decoding accuracy did not change from EARLY to LATE epochs, even as nonshuffled decoding accuracy increased in control tadpoles and decreased in MK-801–treated tadpoles (Figure S9), consistent with a role for neuronal interactions in driving the changes in network performance we observe. These results show that improvements in the brain's ability to represent visual stimuli are not due only to improved encoding in single neurons or local groups, but are driven strongly by changes in the functional organization of the sensory network.

## Discussion

**Figure 9:**
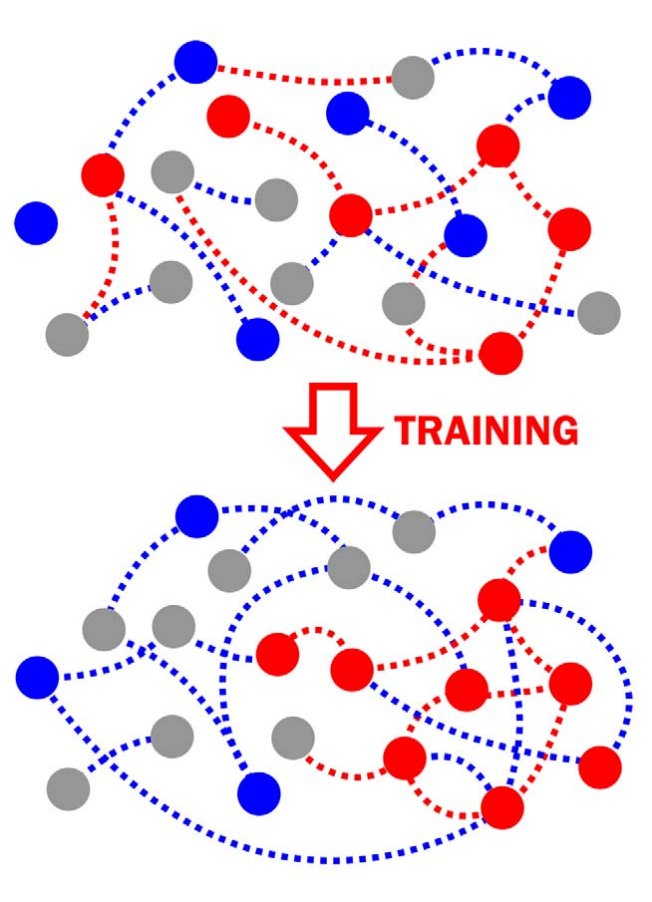
Schematic of receptive field and noise correlation plasticity for trained (red) and untrained (blue) stimuli. Tectal neurons are represented as circles, circle color marks preferred direction (red, down; blue, up), and dotted lines represent noise correlations. Training with down direction increases and clusters receptive fields oriented toward the trained stimuli and decreases long-distance noise correlations (dashed lines). Receptive fields preferring untrained stimuli (blue) are reduced, and noise correlations to these stimuli are increased on all spatial scales. Note that noise correlations can differ across stimuli and are not necessarily determined by neurons' preferred directions.

The functional organization of the brain contributes to effective neural processing, and neurons can coordinate or compete to encode distinct stimulus dimensions [11],[13],[45]. We find that developmental plasticity in response to visual experience establishes such organization in the optic tectum (Figure 9). This plasticity strengthens divisions between microarchitectural brain regions specialized to encode distinct stimuli that the organism experiences. Visual training improves both individual neuron and network response properties, but single-neuron changes only weakly impact network performance. This weak reliance on single neurons likely arises because the tectal network is organized in local receptive field clusters that exhibit high redundancy; information gained from improved fidelity in any individual neuron tends to already be available from other nearby neurons. Our results show that the functional organization of the network plays a larger role in the overall improvement of population encoding with training. This organization consists of specialization by distinct groups of neurons to convey distinct information, as training drives distant neurons to become more independent while strengthening local redundancy. This spatially driven plasticity arises from forces acting to increase or decrease functional connectivity in the tectum on different spatial scales.

Spatial clustering of functional properties is a common feature in the brain [3],[5],[26],[29], which can lead to redundant local encoding. Redundancy is important in mitigating effects of variability of individual neuronal responses. Because neuron response fidelity is fundamentally limited by both physics [46] and physiology [47],[48], redundant encoding by groups can be more practical than decreasing variability in single neurons. Moreover, response properties in a given brain volume are limited by the availability of presynaptic partners, as each neuron must search its local environment for appropriate connections. In tectum, prominent inputs are likely to be shared by nearby neurons because of the localized arborization of retinal ganglion cell axons [40], and plasticity that strengthens those inputs thus promotes local redundancy. Finally, local similarity can make wiring of developing networks more economical [49], as neurons responding to a particular stimulus should then receive inputs from a restricted anatomical region. Learning-associated functional clustering and correlation changes similar to those described here have been described in mouse motor cortex [26], raising the possibility that common constraints drive functional optimization across network structures and functions.

Measurement of single-trial firing rates enables monitoring of redundancy and noise correlations in large populations of tectal neurons. We found that noise correlations can be repeatably measured and are altered by training in an experience- and NMDAR-dependent fashion. These results show that two-photon calcium imaging can be used to investigate shared connections across contiguous brain regions and how these change in vivo. However, the anatomical substrates underlying tectal noise correlation plasticity remain unclear, since noise correlations could arise either from shared retinal inputs or intratectal connections. Plasticity in noise correlations may indicate formation and elimination of these connections or alteration of synaptic strengths. We found that accounting for noise correlations improves decoding of tectal population activity, but this effect could be due to changes in neural activity patterns over the stimulation period [38]. However, the specific effects of NMDAR blockade on noise correlation-based decoding with training suggest that noise correlations are indeed important for decoding tectal activity (Figures 5c and S9). Despite their importance to decoding, we found that the presence of noise correlations does not improve network encoding. The reduction of correlations typically enables networks to convey more information [9]. Indeed, we found that artificially eliminating noise correlations in network activity data increased decoding performance. Networks whose function is limited by the number of neurons available for encoding should thus benefit from decreased noise correlations. Consistent with this prediction, we found that distant network correlations decrease with training in a stimulus-specific manner, as encoding of those stimuli improves. Changes on these larger spatial scales, spanning functional clusters in the tectum, underlie the overall improvement of network encoding with training. Our results show that spatial refinement of noise correlations occurs during experience-dependent plasticity, and changes to such network-level properties are important to the development of tectal function with training.

We find that visual training with motion stimuli induces extensive plasticity in the tectum, distinct components of which are NMDAR dependent or independent. Consistent with previous studies [4],[19],[42], we find no effect of NMDAR blockade on basal motion response properties in tectum. We found that NMDAR-independent mechanisms mediate training-induced increases in reliability and partly mediate improvements in dynamic range, single-neuron mutual information, and neuron-pair mutual information. NMDAR blockade does not completely abolish tectal plasticity [3], and NMDAR-independent plasticity has been described in other systems [50]. However, NMDAR blockade has dramatic effects on coordination of plasticity across the network and components of single-neuron plasticity. When NMDARs are blocked, visual training fails to induce spatially structured changes in tectal network architecture, and NMDAR-independent plasticity drives neurons toward common receptive fields over time. This progressive loss of network organization prevents training from improving whole-network performance. Our findings suggest that NMDARs are essential to coordinated experience-dependent network plasticity by (1) mediating spatial refinement of network connections, leading to localized redundancy and distant correlation reduction, and (2) promoting receptive field diversity and preventing loss of underrepresented receptive fields even as local similarity increases.

Results from training with a restricted stimulus set suggest that competition between synaptic connections underlies network changes in response properties and noise correlations. Training with a subset of stimuli dramatically increased the proportion of responsive neurons with selectivity towards the four stimuli presented, showing that motion-responsive tectal neurons can alter their preferred directions with training, and that stimuli compete for representation by a limited pool of tectal neurons. Furthermore, decreases in noise correlations over the four trained stimuli were accompanied by increases over the untrained stimuli, showing that improvements in stimulus representation can occur at a cost to opposing receptive fields. Training with four stimuli also reduced noise correlations across all spatial distances more dramatically than training with the full eight stimuli, showing that more specific training elicits stronger network plasticity, and suggesting that receptive fields compete for efficient representation by the network.

A number of competitive mechanisms mediated by NMDARs could support the structured plasticity we observe. These mechanisms include removal of axonal projections from tectal regions dominated by opposing axons [40], spike timing-dependent plasticity [25] shown to intrinsically mediate competition between synaptic inputs [51], and NMDAR-dependent metaplasticity [3] that mediates competition by altering plasticity thresholds according to a neuron's overall input rate. Our results demonstrate a role for NMDAR-mediated plasticity mechanisms such as these in experience-driven network refinement.

For developing neurons to form functional networks, each neuron must possess learning mechanisms that change its response properties to ultimately improve whole-network performance. Optimal changes depend on both the specific stimuli encountered and the response patterns of other neurons throughout the network [8],[9],[52]. Our findings show that both of these factors guide NMDAR-dependent plasticity induced by structured visual input in the awake, developing brain.

## Methods

### Animal Rearing Conditions

Freely swimming albino *X. laevis* tadpoles were reared in 0.1× Steinberg's solution (1× Steinberg's in mM: 10 HEPES, 58 NaCl, 0.67 KCl, 0.34 Ca(NO_3_)_2_, 0.83 MgSO_4_, [pH 7.4]) and housed at room temperature on a 12-h light/dark cycle. Experiments were conducted with stage 50 tadpoles in accordance with the Canadian Council on Animal Care guidelines and were approved by the Animal Care Committee of the University of British Columbia Faculty of Medicine.

### Imaging

Oregon Green BAPTA-1 AM (Molecular Probes) was pressure injected into the optic tectum as described previously [3]. 1 h after injection, tadpoles were placed in a bath containing 4 mM pancuronium dibromide for 7 min, then placed in the imaging chamber and immobilized with agar. The imaging chamber was perfused with oxygenated 0.1× Steinberg's solution during imaging. The region imaged was determined by anatomical landmarks and was roughly 200 µm below the surface of the tectum. Images were acquired at 5 Hz using a two-photon laser scanning microscope adapted from an Olympus FV300 confocal microscope (Olympus) and a Chameleon XR Ti:Sapphire laser (Coherent) tuned to 910 nm. Images were acquired using a 60×1.1NA water objective and encompassed a region of roughly 50×150 µm.

### Visual Stimulation

Stimuli were presented on the center of a 6-mm (1,024×768 pixels) LCD screen 7 mm from the surface of the left eye. The screen was covered by a longpass filter to block bleed though of stimulus light into detected fluorescence. Stimuli consisted of solid dark bars with a thickness of 0.09 rad moving at 0.6 rad/s. The edges of the stimulus region were obscured by a circular Gaussian mask, so that the eight stimuli were identical except for rotation and had identical intensity profiles over time. The contrast of stimuli was chosen to be at the threshold of the tadpoles' detection ability, to better compare decoding performance across models over the course of training.

Stimulus presentation and timing were controlled in MATLAB using the Psychophysics Toolbox extensions [53]. Stimuli were presented repeatedly with interstimulus intervals uniform randomly selected from the set (6, 7, 8, 9) s. Movies were acquired in 4-min periods, with 1-min periods for microscope alignment between movies, during which stimuli were shown but images were not recorded. The order of presentation of stimuli was randomized such that an equal number of each stimulus was presented in each 4-min period, and the probability that any stimulus followed any other stimulus was roughly equal over stimulus pairs over the entire experiment.

Tadpoles were presented with one of two stimulus paradigms, 4STIM or 8STIM. Starting 1 h after dye loading, the 4STIM group was presented with a set of four stimuli corresponding to one half of the stimulus space (0–135°) for 1 h, followed by 1 h of the full stimulus space. The 8STIM group was presented with the full stimulus space for 2 h. MK801-treated tadpoles received tectal and ventricular microinjections of 20 µm MK801 after dye loading.

### Two-Photon Guided Patch Recording and Ca^2+^ Imaging

For simultaneous imaging and electrophyisiological recording, loading and imaging of Ca2+ indicators were performed as described above. Tadpoles' heads were mounted in a clear acrylic chamber and held in place by mesh, with tails free to allow respiration. Patch pipettes (tip resistance 7 MOhm), filled with tadpole extracellular solution (115 mM NaCl, 4 mM KCl, 3 mM CaCl2, 3 mM MgCl2, 5 mM HEPES, 10 mM glucose,10 mM glycine; [pH 7.2], adjusted with NaOH; osmolality 255 mOsm) were inserted through the ventricle, approaching the tectum from the medial side. Two-photon imaging was used to guide the pipette tip to responsive neurons and gentle suction was applied to achieve loose seals (80–200 MOhm) at which point action potentials could be clearly discerned. We obtained loose patch recordings at command voltages, which resulted in no net current flow to detect endogenous activity with minimal effect on neuronal firing properties [54]. Imaging and recording were performed while stimulating the contralateral eye with brief flashes from a red LED. Electrical recordings were acquired using an Axon Instruments Axopatch 200B amplifier, digitized at 10 kHz using a Digidata 1322A board, and recorded using pClamp 9 software.

### Fluorescence Data Processing

Fluorescence data stacks were *x*–*y* aligned using Turboreg (ImageJ, NIH) [55]. Experiments that showed vertical drift after alignment were discarded (approximately one in four cases). Custom-written software was used to identify and track regions of interest (ROIs) for each cell over the course of each experiment. Initial ROIs were formed on the basis of morphological characteristics and temporal correlation and excluded cell edges, ensuring no overlapping signal from neighbouring cells. ROIs were then expanded, and these regions were refined and fluorescence signal was denoised using iterated singular value decomposition (SVD), where only pixels with common weighting indicating a positive correlation with cell calcium concentration were retained in successive SVD iterations. Pixels in the expanded region were only retained if they predicted signal in the initial ROI, and if they showed less correlation to overlapping ROIs than the maximum correlation of any pixel in the initial ROI. Raw fluorescence for each cell was the reconstructed time-varying mean pixel intensity based on SVD weightings. The fluorescence time series for each cell was then calculated as (*F*−*F*_0_)/*F*_0_. The time-varying baseline fluorescence, *F*_0_(*t*), was fit for each cell using a Kalman smoother implementing the Rauch-Tung-Striebel algorithm [56]. The model used for the Kalman smoother consisted of a signal with no velocity and Gaussian noise of constant amplitude to model the slowly drifting baseline. The observation of *F*_0_ at each timepoint was the minimum of the smoothed fluorescence trace in a 10-s window around the timepoint, and the covariance was the variance of the raw fluorescence trace within that window, to reflect the confidence that the baseline was observed in that window.

At this point, cells were excluded from the dataset: (1) If fewer than 80% of pixels from the original morphological ROI had common weighting in the SVD decomposition over 80% of the duration of the experiment; this implied that the singular value did not adequately track the calcium concentration of the cell, which should always be positively correlated to fluorescence intensity. (2) If the estimated signal-to-noise ratio for the calcium trace in the cell was less than 1.

Spiking parameters for each cell, including the maximum likelihood spike train, were fit using nonlinear state space methods [30], with initial parameter estimates for spike amplitude, Ca2+ channel time constant, and saturation determined from 10-kHz two-photon imaging line scan data acquired under the same conditions, and fit to each cell using expectation-maximization. After fitting, spike rate time series for each cell were temporally aligned to each other on the basis of *x* and *y* position, to account for the amount of time required to acquire a video frame. Because this model can only place one spike per time bin, it is effective when interspike intervals are consistently longer than the bin width used for inferring spike timings. Over 92% of interspike intervals in electrophysiological recordings during visual stimulation were greater than the 50-ms bin width used for spike inference, and less than 0.1% of time bins contained two spikes, with no bins containing three.

### Single-Neuron Properties

Temporal response curves for each stimulus type were generated by averaging neurons' firing rate in the temporal vicinity of each stimulus over all stimulus presentations of that type. Each neuron's evoked response to each stimulus presentation was the neuron's mean firing rate between an onset and offset latency after the stimulus, which were chosen to maximize the variance of the neuron's activity across stimulus types. Most tadpoles showed potentiation of evoked responses over time (seven of nine 8STIM; seven of nine 8STIM+MK801; three of five 4STIM). Tadpoles showing significant decrease of response amplitude from the first to second hour of training were not included in analyses.

Evoked responses for each neuron were normalized to their mean over each 4-min imaging period to ensure that any changes in overall measured activity would not affect subsequent analyses. Tuning curves were calculated as the mean evoked activity in response to each stimulus over all imaging periods within an epoch of interest. Dynamic range of neuronal tuning curves was defined as the mean absolute deviation of the normalized tuning curve from its mean, 1. Dynamic range is thus the average fraction by which firing rate is altered in response to different stimuli. To compare trained and untrained stimuli in Figure 6, dynamic range was calculated in the same way over each set of four stimuli.

Each neuron's baseline firing rate during each 4-min movie was defined as the median of its spiking rate binned at 200-ms intervals. The 5-s period following each stimulus presentation was excluded from baseline estimation. Neuron reliability in response to each stimulus type was defined as the fraction of stimulus presentations to which the neuron responded with a firing rate greater than baseline.

Orientation and direction selectivity were measured in the manner of Zhang [57]. The centers of the resulting orientation and direction curves, as plotted in Figure 2, were determined by fitting a cos^2^ or angular Gaussian function, respectively. Neurons were considered significantly selective if the amplitude of these fits was significantly different from 0, with variability in initial measurements taken into account. The preferred overall directions plotted in Figure 8 were determined by the 2-D vector sum of neuron-tuning curve values to each direction. The direction of the resulting vector was the preferred orientation.

Single-neuron mutual information is the mutual information between a single neuron's responses and the stimuli, corrected for bias because of limited sample size [58]. For the calculation of mutual information and decoding, evoked activities were discretized into five bins for each neuron, with each bin containing an equal number of samples.

### Network Properties

Neuron-pair mutual information is the mutual information between a bivariate neuronal activity distribution and the stimuli. *p*-Values for bivariate mutual information were estimated by generating random samples with the same number of observations from the independent distribution having the same single-neuron marginal probabilities.

Noise correlation was measured as the correlation between the responses of a pair of neurons to a single stimulus type. With the exception of results presented in Figure 3b and 3c we use mutual information between the two neuron's responses as our measure of correlation, so as not to limit our investigation to linear correlation. In Figure 3b and 3c linear correlations were used to illustrate that these correlations are positive and the nature, not merely the degree, of the correlation is stable.

Tuning curve similarity was defined as the Pearson correlation between tuning curve values across stimuli. To better detect shifts in similarity and because similarity differed across imaging regions, initial similarity was normalized through mean subtraction in each tadpole.

Receptive field diversity is a measure of how well the tuning curves of observed neurons cover the full space of possible receptive fields. We defined this as the variance, across neurons, of tuning curve amplitudes to a given stimulus, summed over all stimuli.

Cooperation in cluster decoding was defined as the decoding performance of two groups taken together minus the maximum decoding performance of either taken alone. Decoding performance is the negative of the mean classification error of the decoder, in degrees. To better display shifts in decoding success with training and under different decoding conditions, decoding performance in Figure 5a and 5c was normalized to initial decoding success of the independent model in each tadpole.

### Decoding Algorithms

Because we do not know the methods that downstream neurons use to decode network information, we build “optimal” decoders—which calculate stimulus probabilities as accurately as possible given an underlying model—so as to measure the overall encoding capability of the network. Decoding of network responses consists of assigning a probability 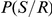 that each stimulus (*S*) was presented, given the network response vector 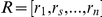, where each *r_i_* is the activity of neuron *i*. The most common approach to this task is to calculate the inverse distribution, 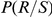, and use Bayes' rule to obtain the desired result:
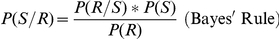
Maximum a posteriori (MAP) decoding consists of identifying the peak of this distribution, useful for categorical classification. These probability distributions are hard to estimate from biological data because the number of neurons, the dimensionality of *R*, is high compared to the number of samples available. A simplifying assumption that is often made is to assume that the firing rates of all neurons are conditionally independent given the stimulus *S*. In this case, 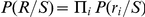. This model requires fewer observations to fit because it requires estimation only of the one-dimensional distribution of *r*_i_ for each stimulus. To perform categorical classification that is sensitive to pairwise interactions between neurons, we used a simple model that relies on the pairwise conditional probability distributions 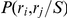, which are more easily estimated than the full distribution but can capture more complexity than the independent model:
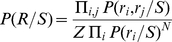
(1)where
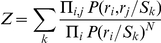
Where the denominator in (1) is a correction for the overrepresentation of single-neuron probabilities in the product of pairwise tables. The optimal value of the parameter *N* depends on the size of the network and its correlation structure. In practice, we selected *N* a priori on the basis of a linear regression of the optimal *N* against sample size in separate test data. A separate regression for *N* was used for cluster decoding presented in Figure 8. A prior probability was added to both models to assure that undersampling would not result in zero probability being assigned to a stimulus-response pair. Parameter settings, i.e., number of bins and prior probability, were chosen to maximize absolute decoding success under the independent model, but results were similar under a wide range of parameter settings. Stimulus probabilities generated by both models were adjusted such that long-run probabilities of all stimuli given the training data were equal.

Assuming sufficiently large samples, this model performs identically to the independent model when neuronal firing is actually independent. For small deviations from independence, consisting of increased probability of a single network pattern, it categorizes stimuli more accurately than the independent model. This decoder outperformed the independent model on virtually all real data we collected, and in artificial datasets of size 3–150 neurons having small pairwise correlations and varying sample sizes (unpublished data). Notably, this model does not make any assumptions about the nature of the bivariate relationships within the network, unlike parametric models such as copulas [59], and allows for graded activity, unlike the Ising model [60].

In all cases, statistics for decoding were calculated from a training set separate from the test set to be decoded, using a “leave-one-out” strategy, in which each short segment (eight stimuli) of activity was decoded using statistics calculated on the basis of all other stimulus presentations in the epoch of interest. For Figure 5c, decoders were trained either on the same epoch being decoded, using a leave-one-out strategy, or all stimulus presentations in the opposite epoch.

Decoding error was defined as the absolute difference between the MAP estimate and the actual stimulus presented, measured in degrees. Decoding improvement is change in decoding error, with positive values representing a decrease in error. Decoding improvement in Figure 5 was measured relative to performance of the independent decoder at the first timepoint.

### Clustering

Clusters (Figure 8c and 8d) were initially formed using the normalized cuts graph clustering algorithm [61] over neuron-pair tuning curve similarity. This was followed by gradient descent to generate groups of uniform size (nine neurons) having maximum within-group similarity. Groups that did not reach a threshold value of within-group similarity were not included in decoding. The group size was made uniform to better compare decoding performance across groups. The number of neurons per group was selected to maximize the difference between the minimum pairwise within-group similarity and the maximum pairwise across-group similarity over all datasets.

The median distance between pairs of neurons within these functionally defined clusters (Figure S8) was used to measure spatial clustering. These medians were compared to the bootstrapped distribution of randomly generated “clusters” using the same neuron positions for each tadpole.

### Statistics

Except where mentioned in the methods and figure captions, unpaired *t*-tests were used to compare mean values across tadpoles.

## Supporting Information

Figure S1 **Methods for fluorescence data processing.** (A) Initial ROIs were identified automatically on the basis of morphological properties and pixel-to-pixel correlations, with cells automatically tracked from video to video. Cells not highlighted drifted out of the imaging plane in one or more videos over the course of the experiment. (B) Expansion of green bounded region in (A). Morphological ROIs are conservative and do not overlap. (C) ROIs are then expanded for spatial filtering using iterated singular value decomposition. (D) Pixel weights indicating the relative contribution of pixels to fluorescence signal reconstruction for their respective cells. Brighter pixels indicate higher weighting. (E) Time-varying baseline fluorescence (*F*_0_) was fit using a Kalman filter smoother taking into account the amplitude of spiking to estimate the accuracy of baseline estimates.
(JPG)

Figure S2 **Correlation of optical and electrophysiological firing rate measurements.** Left, simultaneous recording of somatic fluorescence (Δ*F*/*F*_0_, top) and action potentials (green) in response to full field light stimuli of varying intensity, with actual (gray) and inferred (black) firing rates in the 5 s following each stimulus, for three different cells. Right, expanded voltage traces for the regions marked in red at left. Pink shading marks time of stimulus. The electrical transients bounding the stimulus period are clipped. Colored dots mark individual action potentials, which are magnified in the boxes at bottom.
(JPG)

Figure S3 **Optical measures of firing rate are correlated with electrophysiological measurements.** Left, scatterplots of number of spikes evoked by visual stimuli versus inferred firing rate (b) measured optically. Evoked spikes refers to total number of spikes evoked in the 5-s period following stimulus onset. Each point represents a single stimulus presentation, and symbol colors correspond to distinct neurons. All optical recording parameters (duration, frame rate, optical setup) and fitting method for spike inference were identical to experiments performed with optical methods alone. Right, correlation between visually evoked firing rates obtained from cell-attached recording and (left) inferred firing rates or (right) peak Δ*F*/*F*_0_. Firing rate inference outperformed peak Δ*F*/*F*_0_ (paired *t*-test, *p* = 0.001). *n* = 5 visually responsive neurons.
(JPG)

Figure S4 **Noise correlations differ across stimuli.** Distribution of magnitude of Fisher's z (normalized to expected SD) for all pairwise comparisons of noise correlation coefficients in neuron pairs. Dotted line represents the null distribution (normal with unit variance). Observed noise correlations between neuron pairs vary across stimuli 14% more than expected by chance if they were actually equal (*p*<10^−12^; Chi-square variance test).
(JPG)

Figure S5 **Noise correlation encoding.** Responses of two example neurons to two stimulus types. Arrows denote the two stimulus directions plotted. As their single-neuron firing distributions (top and right) indicate, neither neuron taken alone significantly discriminates the two stimuli. However, because noise correlations differ for the stimuli, the joint firing distribution (center) does discriminate them: when presented with a left moving stimulus (blue), neuron 2 is strongly active only when neuron 1 is inactive (negatively correlated); when presented with a right moving stimulus (red), neuron 2 is strongly active only when neuron 1 is strongly active (positively correlated). As discussed in the text, such encoding is not prominent in the tectum.
(JPG)

Figure S6 **Receptive field similarity and noise correlation are associated.** (**a**) Scatterplot of signal correlation versus mean linear (Pearson's) noise correlation between tectal neuron pairs. Black points fall outside two SDs of mean of the null distribution. (**b**) Quantification of (**a**). Mean signal correlation binned for extreme (>two SDs from the mean) and moderate noise correlations. Values are mean ± standard error of the mean (SEM).
(JPG)

Figure S7 **The noncompetitive NMDA receptor antagonist MK-801 blocks evoked NMDA receptor currents in** ***Xenopus*** **tectal neurons in vivo.** Whole cell patch clamp recordings were performed at a holding potential of +55 mV while stimulating axonal inputs at the optic chiasm in the presence of CNQX (10 m) to block AMPA receptor currents. Addition of 20 M MK-801 caused a progressive blockade of evoked synaptic NMDA receptor mediated currents. Colors denote recording trials before (black), and the first, tenth, and 29th stimulation trials after MK-801 application, with a 10-s interstimulus interval. Complete blockade of NMDA receptor-mediated currents were observed in a total of five neurons recorded from five tadpoles.
(JPG)

Figure S8 **Neuron receptive fields are spatially clustered.** Median neuron-neuron distance within groups generated by the clustering algorithm, which is based only on tuning curves. This is the median distance between pairs of neurons belonging to the same group, averaged across all groups in a given tadpole. Values are the mean ± SEM over *n* = 7 tadpoles (29 clusters). Dotted line is the mean value of this measure across 1,000 randomly selected “clusters” in each tadpole using the same neuron positions that were included in the real clusters. Neurons with similar receptive fields are closer to each other than expected by chance (two-sample I-test). *p<0.05; **p<0.01.
(JPG)

Figure S9 **Performance of shuffled decoders does not change with training.** Performance of decoders trained and tested on shuffled (blue) or unshuffled (orange) data during early (left) and late (right) epochs in control (top) and MK-801–treated (bottom) tadpoles. To generate shuffled data, responses to each stimulus type were shuffled for each neuron, a procedure that removes noise correlations but maintains neuronal tuning curves. Asterisks denote significant difference relative to the same decoder in the early epoch (paired *t*-test). **p<0.01.
(JPG)

## Abbreviations

NMDAR: N-methyl-D-aspartic-acid type glutamate receptor
ROI: regions of interest
SEM: standard error of the mean
SVD: singular value decomposition
