# Supplementary figures and images for: Functional Clustering Drives Encoding Improvement in a Developing Brain Network during Awake Visual Learning
Source: PLoS Biol. 2012 Jan 10;10(1):e1001236. doi: 10.1371/journal.pbio.1001236 (PMC3254648; doi:10.1371/journal.pbio.1001236)

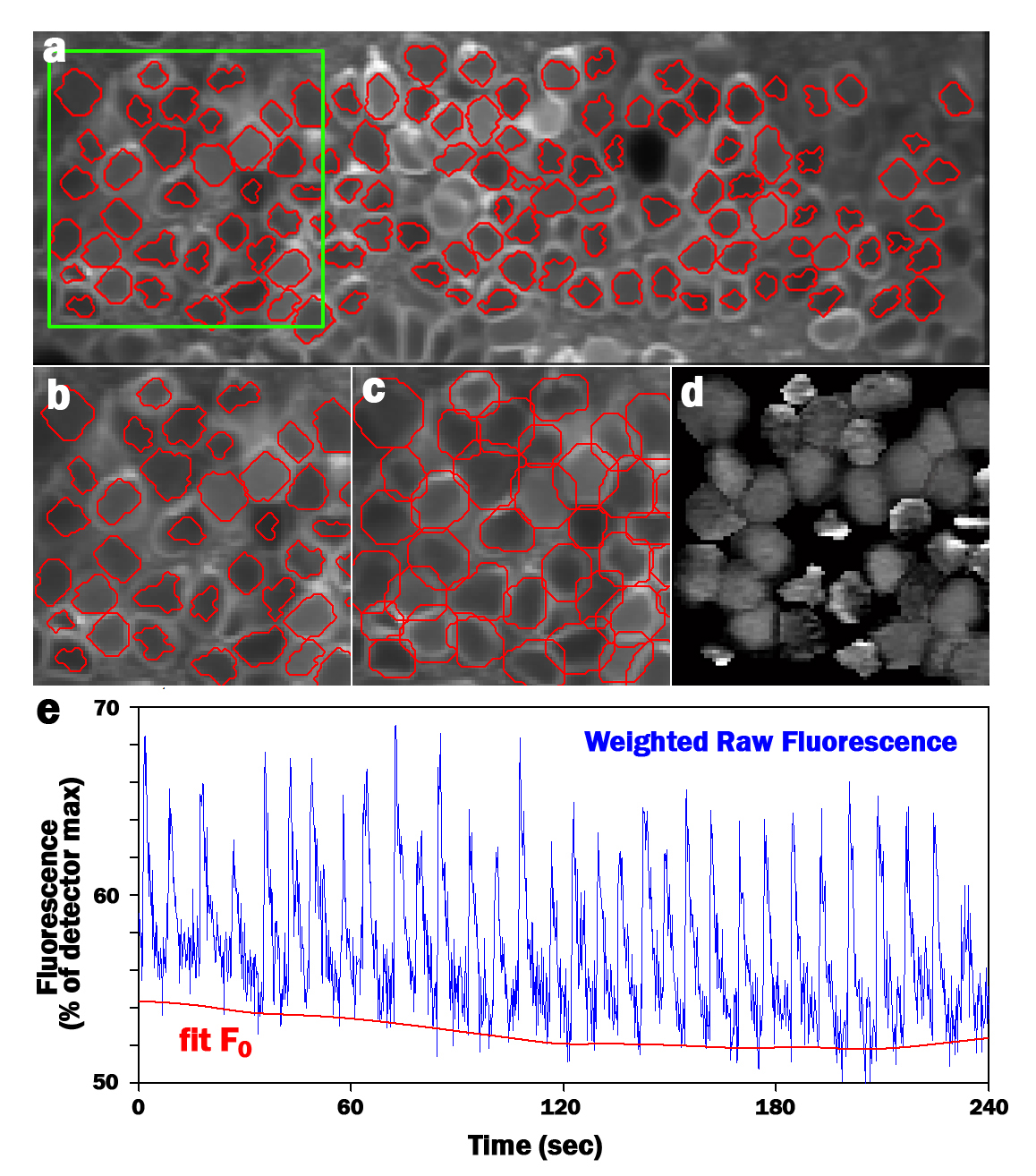

Supplement: Figure S1 — Methods for fluorescence data processing. (A) Initial ROIs were identified automatically on the basis of morphological properties and pixel-to-pixel correlations, with cells automatically tracked from video to video. Cells not highlighted drifted out of the imaging plane in one or more videos over the course of the experiment. (B) Expansion of green bounded region in (A). Morphological ROIs are conservative and do not overlap. (C) ROIs are then expanded for spatial filtering using iterated singular value decomposition. (D) Pixel weights indicating the relative contribution of pixels to fluorescence signal reconstruction for their respective cells. Brighter pixels indicate higher weighting. (E) Time-varying baseline fluorescence (F 0) was fit using a Kalman filter smoother taking into account the amplitude of spiking to estimate the accuracy of baseline estimates. (JPG) [file pbio.1001236.s001.jpg]

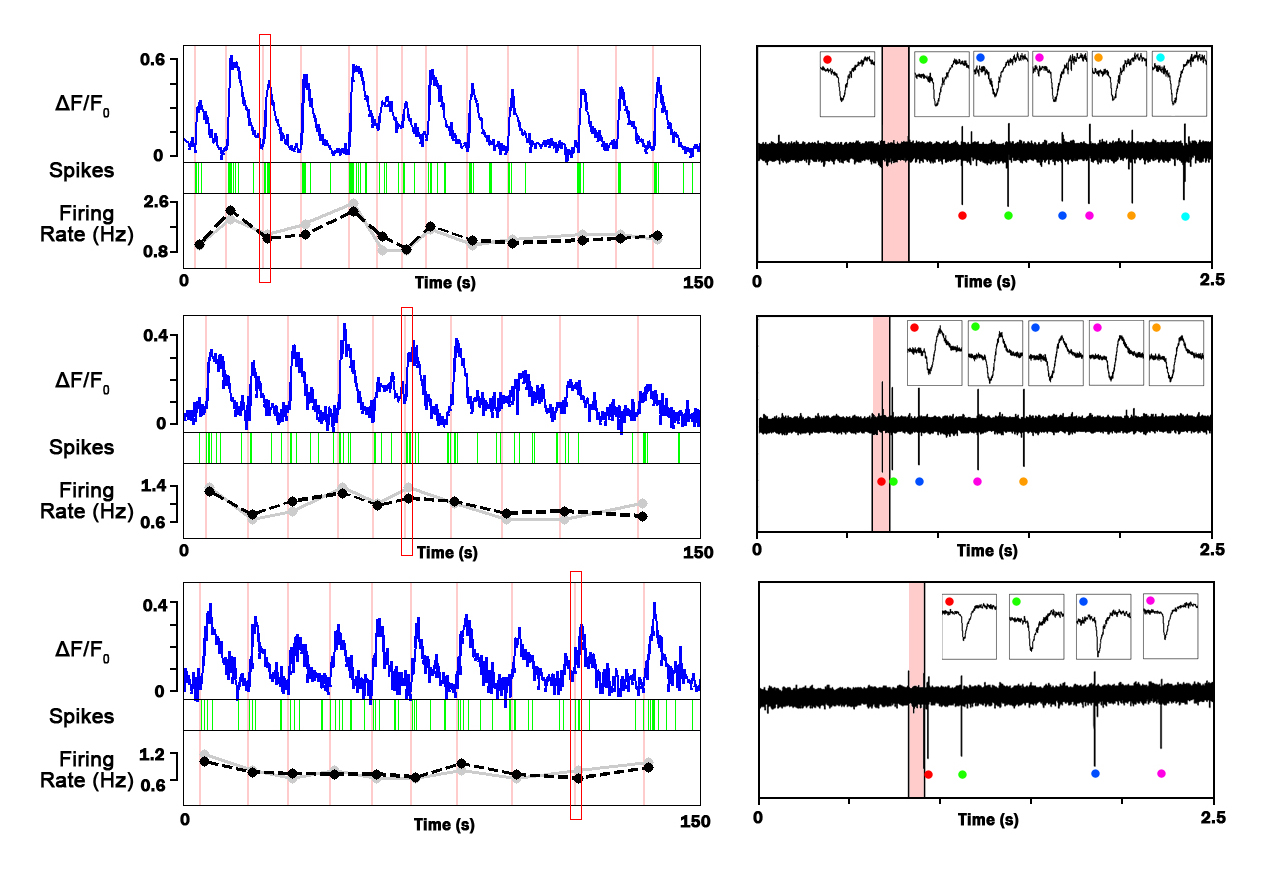

Supplement: Figure S2 — Correlation of optical and electrophysiological firing rate measurements. Left, simultaneous recording of somatic fluorescence (ΔF/F 0, top) and action potentials (green) in response to full field light stimuli of varying intensity, with actual (gray) and inferred (black) firing rates in the 5 s following each stimulus, for three different cells. Right, expanded voltage traces for the regions marked in red at left. Pink shading marks time of stimulus. The electrical transients bounding the stimulus period are clipped. Colored dots mark individual action potentials, which are magnified in the boxes at bottom. (JPG) [file pbio.1001236.s002.jpg]

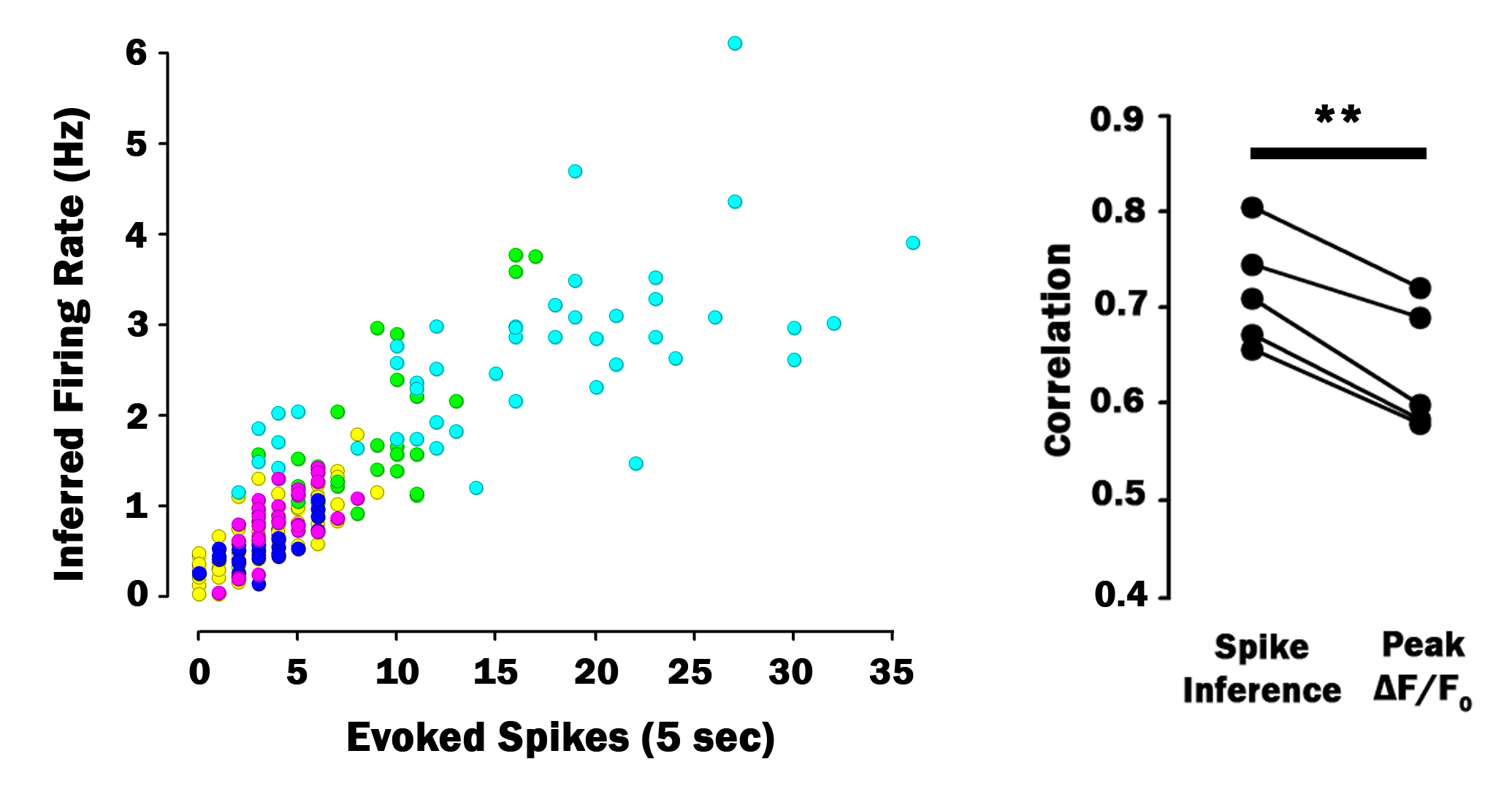

Supplement: Figure S3 — Optical measures of firing rate are correlated with electrophysiological measurements. Left, scatterplots of number of spikes evoked by visual stimuli versus inferred firing rate (b) measured optically. Evoked spikes refers to total number of spikes evoked in the 5-s period following stimulus onset. Each point represents a single stimulus presentation, and symbol colors correspond to distinct neurons. All optical recording parameters (duration, frame rate, optical setup) and fitting method for spike inference were identical to experiments performed with optical methods alone. Right, correlation between visually evoked firing rates obtained from cell-attached recording and (left) inferred firing rates or (right) peak ΔF/F 0. Firing rate inference outperformed peak ΔF/F 0 (paired t-test, p = 0.001). n = 5 visually responsive neurons. (JPG) [file pbio.1001236.s003.jpg]

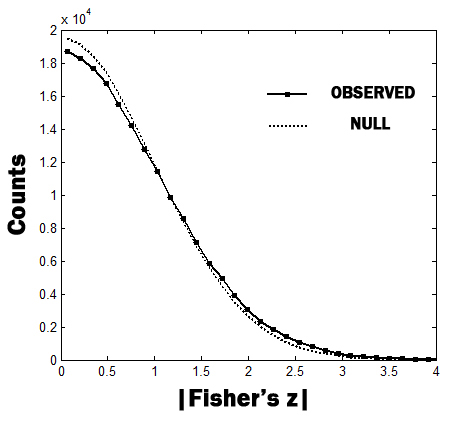

Supplement: Figure S4 — Noise correlations differ across stimuli. Distribution of magnitude of Fisher's z (normalized to expected SD) for all pairwise comparisons of noise correlation coefficients in neuron pairs. Dotted line represents the null distribution (normal with unit variance). Observed noise correlations between neuron pairs vary across stimuli 14% more than expected by chance if they were actually equal (p<10−12; Chi-square variance test). (JPG) [file pbio.1001236.s004.jpg]

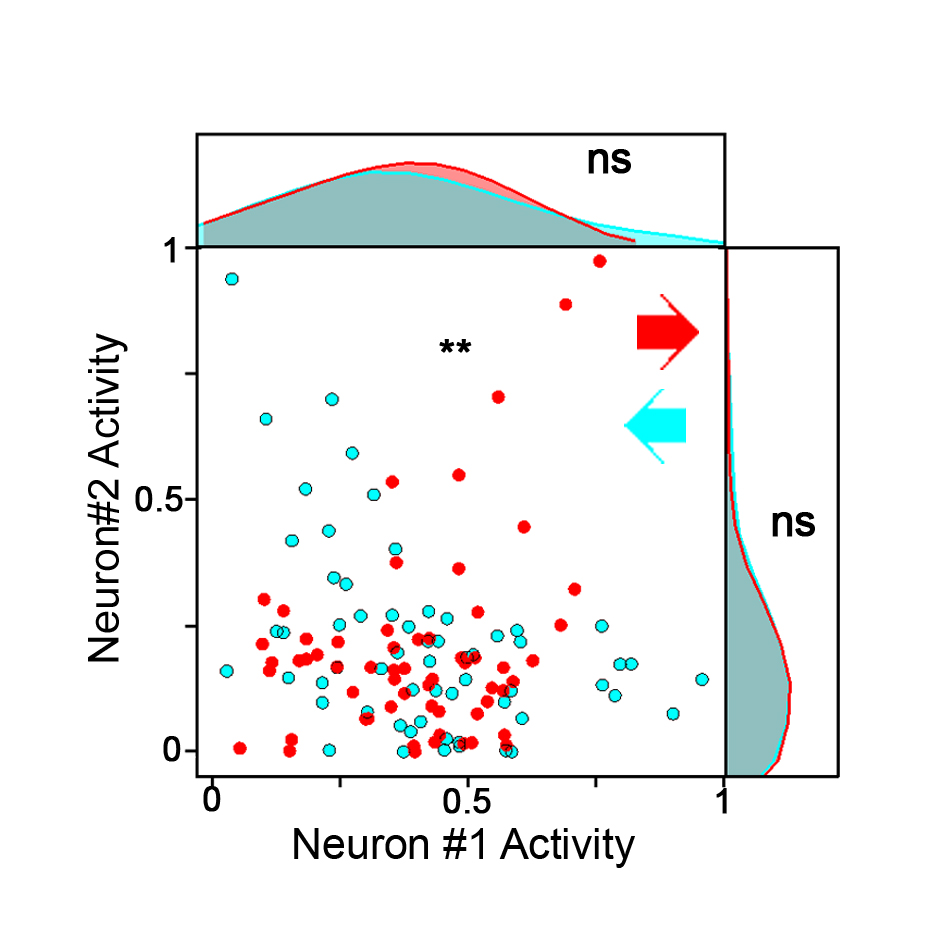

Supplement: Figure S5 — Noise correlation encoding. Responses of two example neurons to two stimulus types. Arrows denote the two stimulus directions plotted. As their single-neuron firing distributions (top and right) indicate, neither neuron taken alone significantly discriminates the two stimuli. However, because noise correlations differ for the stimuli, the joint firing distribution (center) does discriminate them: when presented with a left moving stimulus (blue), neuron 2 is strongly active only when neuron 1 is inactive (negatively correlated); when presented with a right moving stimulus (red), neuron 2 is strongly active only when neuron 1 is strongly active (positively correlated). As discussed in the text, such encoding is not prominent in the tectum. (JPG) [file pbio.1001236.s005.jpg]

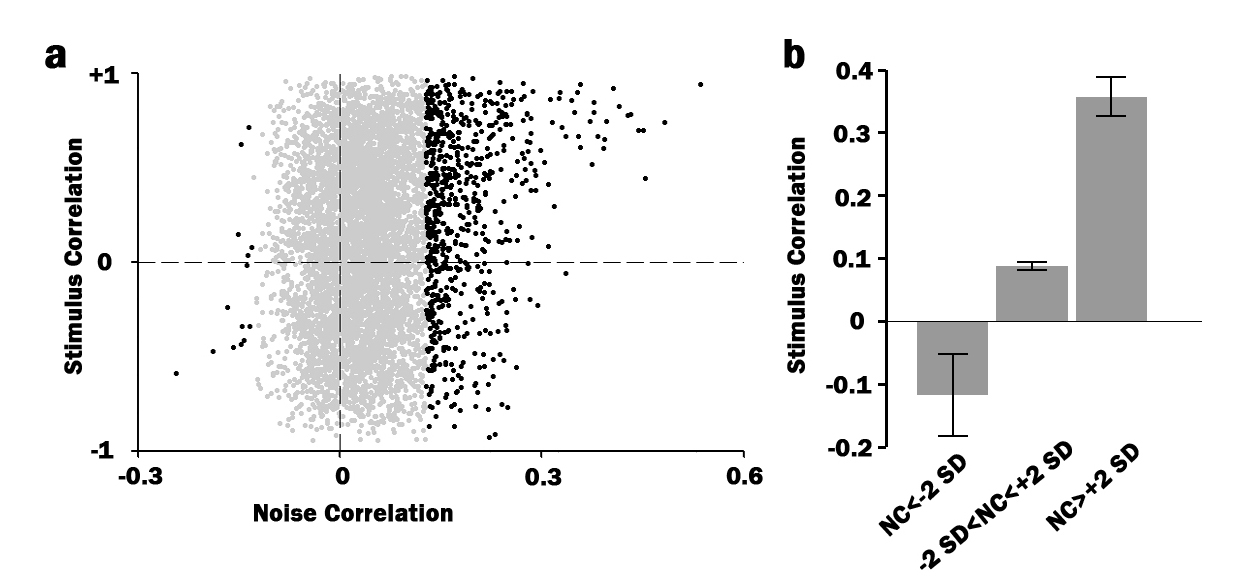

Supplement: Figure S6 — Receptive field similarity and noise correlation are associated. (a) Scatterplot of signal correlation versus mean linear (Pearson's) noise correlation between tectal neuron pairs. Black points fall outside two SDs of mean of the null distribution. (b) Quantification of (a). Mean signal correlation binned for extreme (>two SDs from the mean) and moderate noise correlations. Values are mean ± standard error of the mean (SEM). (JPG) [file pbio.1001236.s006.jpg]

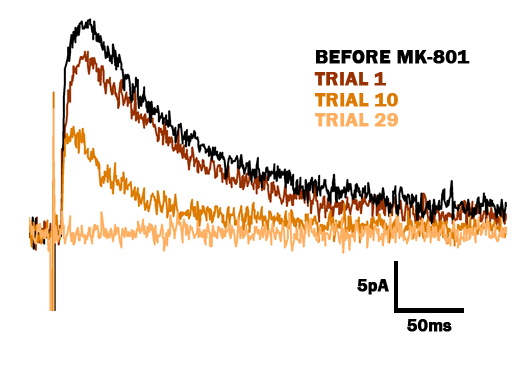

Supplement: Figure S7 — The noncompetitive NMDA receptor antagonist MK-801 blocks evoked NMDA receptor currents in Xenopus tectal neurons in vivo. Whole cell patch clamp recordings were performed at a holding potential of +55 mV while stimulating axonal inputs at the optic chiasm in the presence of CNQX (10 m) to block AMPA receptor currents. Addition of 20 M MK-801 caused a progressive blockade of evoked synaptic NMDA receptor mediated currents. Colors denote recording trials before (black), and the first, tenth, and 29th stimulation trials after MK-801 application, with a 10-s interstimulus interval. Complete blockade of NMDA receptor-mediated currents were observed in a total of five neurons recorded from five tadpoles. (JPG) [file pbio.1001236.s007.jpg]

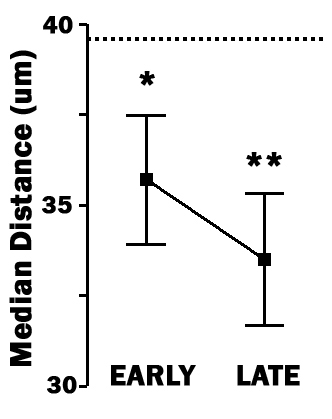

Supplement: Figure S8 — Neuron receptive fields are spatially clustered. Median neuron-neuron distance within groups generated by the clustering algorithm, which is based only on tuning curves. This is the median distance between pairs of neurons belonging to the same group, averaged across all groups in a given tadpole. Values are the mean ± SEM over n = 7 tadpoles (29 clusters). Dotted line is the mean value of this measure across 1,000 randomly selected “clusters” in each tadpole using the same neuron positions that were included in the real clusters. Neurons with similar receptive fields are closer to each other than expected by chance (two-sample I-test). *p<0.05; **p<0.01. (JPG) [file pbio.1001236.s008.jpg]

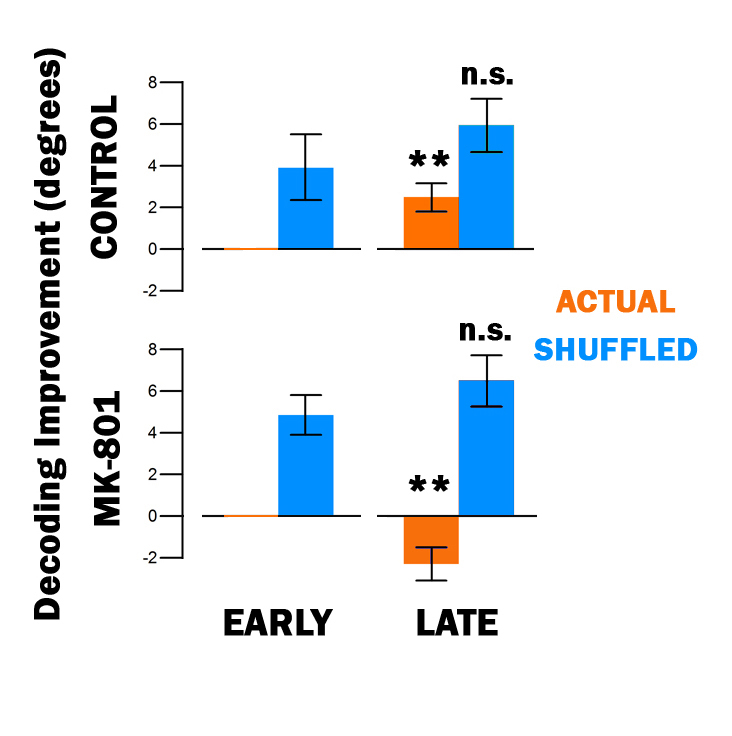

Supplement: Figure S9 — Performance of shuffled decoders does not change with training. Performance of decoders trained and tested on shuffled (blue) or unshuffled (orange) data during early (left) and late (right) epochs in control (top) and MK-801–treated (bottom) tadpoles. To generate shuffled data, responses to each stimulus type were shuffled for each neuron, a procedure that removes noise correlations but maintains neuronal tuning curves. Asterisks denote significant difference relative to the same decoder in the early epoch (paired t-test). **p<0.01. (JPG) [file pbio.1001236.s009.jpg]
